# Beyond the Cell: Stem Cell‐Derived Exosomes as Next‐Generation Tools in Regenerative Therapy

**DOI:** 10.1155/sci/1054968

**Published:** 2026-10-07

**Authors:** Hazem A. El-Kady, Majdeldin E. Abdelgilil, Hagar W. Mandour, Rahaf Altartoor, Ziad Mustafa Abd El-Wahab, Sarah Alosman, Soha M. Abdelzaher, Ibrahim M. Fangary, Yasmine Hosny

**Affiliations:** ^1^ Applied Biotechnology Program Faculty of Science Ain Shams University Cairo Egypt asu.edu.eg; ^2^ Molecular Biology and Biotechnology Department School of Biotechnology Badr University in Cairo Cairo Egypt buc.edu.eg; ^3^ Biotechnology Department Faculty of Science King Salman International University South Sinai Egypt ksiu.edu.eg; ^4^ Department of Molecular Biology and Genetics Istanbul Medeniyet University Istanbul Türkiye medeniyet.edu.tr; ^5^ Zoology Department Faculty of Science Ain Shams University Cairo Egypt asu.edu.eg

**Keywords:** characterization, exosomes, isolation, molecular cargo, regenerative medicine, stem cells, therapeutic applications

## Abstract

Exosomes derived from stem cells have gained significant interest in recent years due to their considerable promise as a cell‐free therapy in drug delivery and regenerative medicine. Their therapeutic appeal lies in their immune‐modulating properties, along with a lower risk of immune response or tumor formation compared to whole cell‐based therapies. It has been highlighted that exosomes can be engineered to deliver therapeutic molecules such as RNAs, proteins, and drugs to specific cells, tissues, and organs, making them a promising tool for targeted therapies. This review summarizes the sources of exosomes from different types of stem cells, covering the components of their molecular cargos from different types of RNAs, proteins, growth factors, and other molecules. The focus is on the different advanced methods used to isolate and characterize the exosomes. Exosomes have many therapeutic applications related to cancer, cardiovascular diseases, neurodegenerative disease, wound healing, bone repair, and renal and hepatic injuries.

## 1. Introduction

Cells secrete and release many types of membrane‐bound vesicles into the extracellular environment. These extracellular vesicles (EVs) originate either directly from the plasma membrane or from the endosomal system. Exosomes are a specific subset of EVs characterized by their small size, typically ranging from 30 to 150 nanometers in diameter. However, the specific size ranges reported for exosomes from different stem cell types vary somewhat across studies. This variability reflects genuine biological heterogeneity among vesicle populations from different cellular sources, as well as differences in isolation technique. Unlike other types of EVs such as microvesicles or apoptotic bodies, exosomes originate from the endosomal pathway inside the cell [1].

The biogenesis of exosomes begins when the plasma membrane buds inward to form early endosomes. As these mature into late endosomes, their limiting membrane invaginates, generating intraluminal vesicles (ILVs) within larger structures known as multivesicular bodies (MVBs). Fusion of MVBs with the plasma membrane then releases the ILVs into the extracellular space as exosomes [2] (Figure 1).

**Figure 1:**
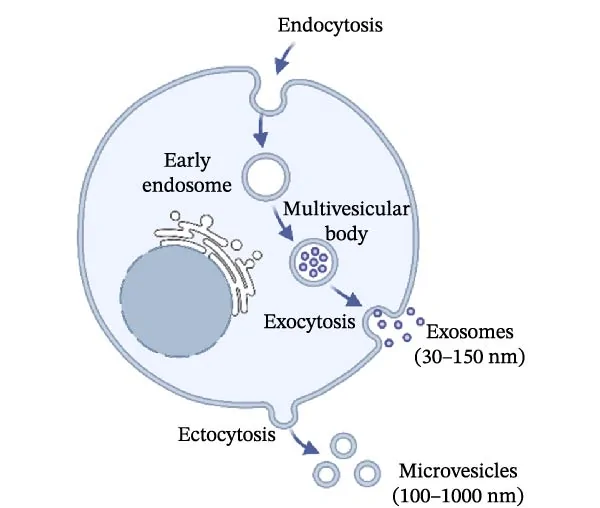
Biogenesis of exosomes through endosomal pathways. This figure was created by the authors with Biorender.com.

Exosomes carry a rich cargo of biological molecules, including proteins, lipids, and various types of nucleic acids (such as mRNA, microRNA, and DNA fragments), which reflect the cell type they originate from and its physiological state. The molecular content is selectively packaged, enabling exosomes to function as messengers in cell‐to‐cell communication, influencing processes such as immune responses, tissue repair, and even tumor progression [3].

Their lipid bilayer membrane is enriched with specific lipids, such as cholesterol, sphingomyelin, and ceramides, which contribute to their stability and ability to fuse with recipient cells. Surface proteins, including tetraspanins (CD9, CD63, and CD81), heat shock proteins, and major histocompatibility complex (MHC) molecules, serve as markers for exosome identification and play roles in targeting and uptake by recipient cells [4, 5].

Exosomes are released by most cell types under both normal and diseased conditions and can be isolated from various body fluids, including blood and malignant effusions. They play critical roles in intercellular communication, mediating processes such as nonclassical protein secretion, antigen presentation, signal transduction, and the transfer of RNA and proteins [4].

In disease states, exosomes mirror pathological tissue alterations and are readily detectable in the body fluids. This makes them powerful tools for both diagnostics and therapeutics, within a rapidly evolving field poised to transform disease management [6–8].

## 2. Sources of Stem Cells‐Derived Exosomes

Stem cell‐derived exosomes function similarly to their parent cells; moreover, they have advantages over cell‐based therapy, especially the ability to avoid concerns such as immunological rejection and tumorigenicity. Exosomes can be used in conjunction with current techniques to make carrier particles, support stem cell paracrine function, and transfer information across cells. They can also have automated targeting abilities and be designed to target certain cells or tissues. Because of these characteristics, exosomes are generally considered to carry a lower risk of tumor formation than cell‐based therapy, which makes them ideal for the development of nanomedicine [9].

Although exosomes from different stem cell types share a common biogenesis pathway and core tetraspanin markers, the identity, potency, and clinical accessibility of their parental cells differ substantially, which in turn shapes the exosome cargo and translational profile (Tables 1 and 2).

**Table 1:** Comparative characteristics of different types of stem cells.

Feature	ESCs	iPSCs	MSCs	NSCs
Origin	Blastocyst inner cell mass	Reprogramed adult somatic cells	Bone marrow, adipose, cord tissue, etc.	Subventricular/subgranular neurogenic niches
Potency	Pluripotent	Pluripotent	Multipotent	Multipotent (neural lineages)
Ethical constraint	Ethical concerns; allogeneic rejection	Reprograming and genomic‐stability risk	None major; widely accessible	Limited accessibility in vivo
Tumorigenic risk of parent cells	High (teratoma)	High (teratoma)	Low	Low
Clinical translation stage of exosomes	Preclinical	Preclinical	Most advanced/early clinical trials	Preclinical
References	[10–13]	[14–20]	[21–30]	[31–33]

**Table 2:** Comparative characteristics of exosomes from different example sources.

Type	Size (nm)	Key surface markers	Cargo contents	Biological effects	Mechanisms of action	Advantages over parent cells	References
MSC‐derived exosomes	50–200	CD63, CD9, CD81	miRNAs (e.g., miR‐21, miR‐181 c), VEGF, MMPs, heat shock proteins	Angiogenesis, anti‐inflammatory, immune modulation	Paracrine signaling, modulate Wnt/MAPK, carry VEGF, MMPs	Avoid immune rejection, programable targeting, safer	[9, 34]
ESC‐derived exosomes	50–150	CD63, CD9, TSG101, Alix [8]	Proteins (FGF2, HLA‐G), mRNAs (Oct‐4, SOX2, KLF4, NANOG), miR‐132	Maintain pluripotency, mesodermal/cardiomyocyte differentiation	EVs synchronize cell phenotype, activate PKA/FAK	Facilitate communication without direct cell use	[10, 35]
IPSC‐derived exosomes	30–160	CD63, CD9, CD81, SSEA‐4, CD133−1, CD326	miRNAs (e.g., miR‐21, miR‐210), pluripotency markers	Cardioprotection, autologous repair, pluripotency support	Deliver miRNAs, suppress apoptosis, endogenous repair	Autologous therapy, safer than stem cells	[14, 36]
NSC‐derived exosomes	30–200	CD63, CD81, ICAM‐1, MFG‐E8, TSG101, ALIX	miRNAs (e.g., miR‐210, miR‐9), mRNA, lipids, proteins	Neuro regeneration, synaptic regulation, neurovascular integrity	miRNA‐mediated (e.g., miR‐320‐5p, miR‐210/Hes1 axis)	Non‐tumorigenic, cross BBB, scalable, low immunogenicity	[31, 33]

*Note*: CD9, CD63, and CD81 are general EV/exosome identity markers shared across virtually all cell types and are not source‐specific; the distinct biological effects listed for each exosome type derive from differences in the broader miRNA, protein, and lipid cargo rather than from these shared surface markers.

### 2.1. ESCs

Embryonic stem cells (ESCs) are pluripotent cells, which can differentiate into almost any cell type. The ESCs are specifically found in the inner cell mass (ICM) of blastocyst‐stage embryos [10].

Exosomes derived from ESCs are characterized by their size range, typically falling within 50–150 nm, with a distinct ball‐shaped morphology [11]. Exosomal markers such as CD63, CD9, TSG101, and Alix are used in the verification and characterization of ESC exosomes. They also contain hundreds of proteins; the most abundant are fibroblast growth factor (FGF2) and human leukocyte antigen‐G (HLA‐G). ESC‐derived exosomes also contain pluripotency‐associated mRNAs, including SOX2, OCT4, NANOG, and KLF4, reflecting the biological phenotype of the parent ESCs [11–13].

### 2.2. IPSCs

Induced pluripotent stem cells (IPSCs) are adult (somatic) cells that have been reprogramed back into a pluripotent state, making them functionally similar to ESCs (Figure 2). Yamanaka factors (Oct3/4, Sox2, Klf4, and c‐Myc) could rewire genetically differentiated mature cells into an embryonic undifferentiated state using retroviral vectors [14, 15].

**Figure 2:**
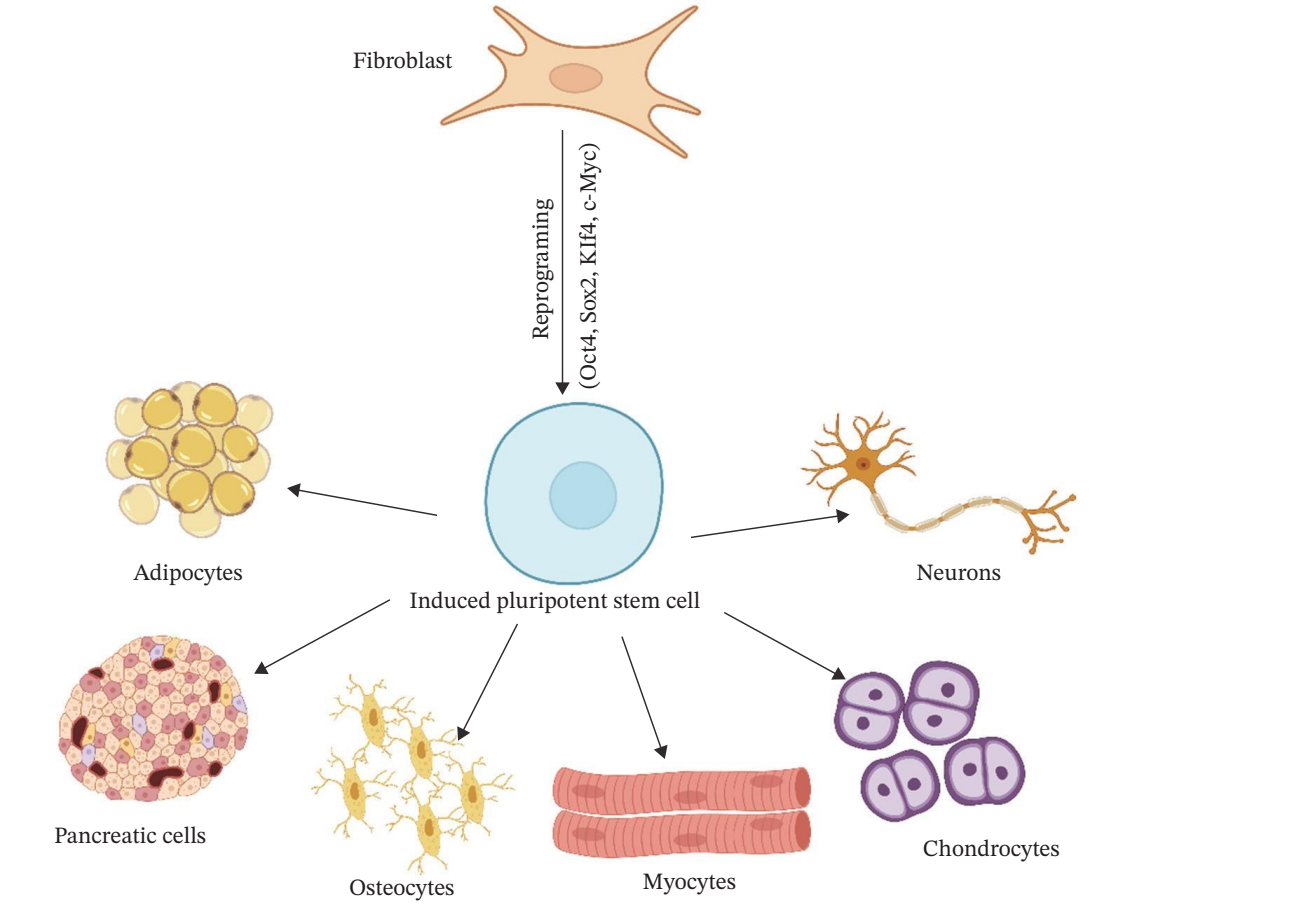
Generation and multilineage differentiation potential of induced pluripotent stem cells (IPSCs). This figure was created by the authors with BioRender.com.

Exosomes derived from induced pluripotent stem cells (iPSC‐Exos) are highly similar to those derived from ESCs. They typically exhibit a size range of ~30–160 nm and a spherical morphology. iPSC‐Exos express classical exosomal surface markers, including the tetraspanins CD9, CD63, and CD81. In addition, pluripotency‐associated markers such as SSEA‐4, CD133−1 (Prominin‐1), CD326 (EpCAM), CD24, and receptor tyrosine kinase‐like orphan receptor 1 (ROR1) may be present and can be utilized for the characterization of iPSC‐derived exosomes [16–19].

Since the use of IPSCs in regenerative medicine has been somewhat limited by their tumorigenic potential, the use of IPSC‐exosomes has served as a successful alternative for cell‐free therapies, minimizing the risk of an immune response [19]. Furthermore, by imitating endogenous repair, exosomes from IPSCs can facilitate autologous therapy. Therefore, compared to stem cell transplantation, the exosomes produced from patient‐specific IPSC‐derivatives should offer greater therapeutic specificity, ensuring quality, safety, and ease of manufacture [20].

### 2.3. Adult Stem Cells

Adult stem cells, also known as somatic stem cells, are undifferentiated cells found in a variety of tissues throughout the body, including skin, heart, brain, liver, and bone marrow. Unlike ESCs, which can specialize to any cell type in the body, adult stem cells are typically restricted to becoming any type of cell in the tissue or organ in which they reside. Among adult stem cells, mesenchymal stem cells (MSCs) are the most widely studied and utilized due to their accessibility, multipotency, and immunomodulatory properties, making them ideal candidates for regenerative medicine, cell therapy, and tissue engineering [21, 23].

MSC‐derived exosomes are nanosized spheres ranging from 50 to 200 nm. The composition of MSC‐derived exosomes along with expressing the markers CD63, CD9, and CD81 [24], have a number of miRNAs, such as miR‐191, miR‐222, miR‐21, and let‐7a, which affect cell proliferation and angiogenesis, and miR‐6087 that facilitates the differentiation of endothelial cells. Heat‐shock proteins (Hsp60, Hsp70, and Hsp90), MHC class I and II molecules, GTPases, and other proteins involved in multivesicular body biogenesis, such as Alix and TSG101. They also carry cytoskeletal proteins (actin, moesin, and syntenin) and anti‐inflammatory cytokines (TGF‐β1, IL‐6, IL‐10, and HGF) that play a role in regulation of inflammation and immune modulation [25].

MSC‐exosomes have several mechanisms to work with such as paracrine signaling and cargo transfer. For example, exosomes deliver miRNAs (like miR‐19a, miR‐21, and miR‐16) that regulate gene expression in recipient cells, such as suppressing apoptosis‐related genes or angiogenesis inhibitors, thus promoting repair and regeneration [26–29].

Specifically, MSC‐derived exosomes do not have a fixed content; rather, their composition is shaped by the tissue source, cellular activities, and surrounding cells. Notably, the exosomal content is altered when MSCs are co‐cultured with tumor cells or placed within a tumor microenvironment, indicating that exosomes actively reflect the physiological and pathological conditions of their origin [30].

Neural stem cells (NSCs) represent another important type of adult stem cells, responsible for maintaining and regenerating neurons and glial cells in the nervous system. The NSCs are found in neurogenic niches, which consist of the subventricular zone (SVZ) of the lateral ventricles and the subgranular zone (SGZ) in the hippocampus’s dentate gyrus [31, 32].

Neural exosomes can be identified by its unique cargo. Tetraspanins, heat shock proteins, membrane transport and fusion proteins, phospholipases, and lipid‐related proteins are some of the examples. The proteins AIP1/ALIX, TSG101, ICAM‐1, MFG‐E8, 1‐integrin, CD81, and CD63 are among those present in exosomes. Exosomes frequently contain the following lipids: phosphatidylcholine, sphingomyelin, cholesterol, and phosphatidylserine and phosphatidylinositol [33].

Exosomes secreted by NSCs are associated in neurons and microglia interactions in the CNS. They have the capability to transport active compounds from neurons to astrocytes, potentially regulating myelination, synaptic activity, astrocyte function, and neurovascular integrity. Exosomes from NSCs are high in certain miRNAs that can mediate viral entrance into cells, communicate with the microenvironment, and serve independent metabolic units [37].

In cases such as traumatic brain injury (TBI), a variety of cross‐reactions allow NSCs to perform endogenous brain repair, including vascular regeneration and neurogenesis. In this process, exosomes produced from NSCs play a crucial role as signaling molecules that speed up tissue healing and mediate physiological processes. Moreover, exosomes generated from NSCs improve the vitality, proliferation, and neuronal brain growth of newborns [33].

The NSC exosomes can be administered intranasally so lots of them can be directed to various areas of the brain. They rapidly disintegrate after releasing the cargo, substantially reducing their susceptibility to tumor development or malignant transformation relative to cell therapy, although this has not been definitively established for all exosome preparations. They can also be administered intravenously without developing thrombosis, or blockage of small blood arteries, because they are small vesicles that can cross the blood–brain barrier (BBB) and transcytose via endothelial cells.

Large‐scale production can now be employed to produce therapeutic vesicles. Furthermore, EVs are easy to store and transport since they maintain stable for several weeks at 4°C and for extended periods of time at −80°C. However, because stem‐cell‐secreted EVs may show some risky characteristics in certain conditions, EVs must be thoroughly characterized for consistency in their composition and biological effects before being clinically translated to prevent any adverse reactions. Therefore, EVs that offer cell‐free therapy have the potential to transform medicine and save a lot of lives without causing serious adverse effects [33].

## 3. Methods of Exosome Isolation

### 3.1. Conventional Exosome Isolation Techniques

The discovery of exosomes has transformed the understanding of cellular communication, providing a groundbreaking capability in scientific innovation, personalized medicine, and biotechnology. However, their isolation and characterization have consistently posed challenges in maintaining the integrity of exosomes (Table 3). The heterogeneous environment of lipid bilayer vesicles comprises exosomes, microvesicles, and apoptotic bodies, each possessing different sizes and characteristics, making a universally standard isolation method nonexistent [38, 51]. These EVs can be separated based on their biophysical characteristics using several techniques, such as ultracentrifugation, size‐based method (ultrafiltration), and precipitation [38, 51]. The technique is chosen depending on the origin of the exosomes, the required purity, and their downstream application [39].

**Table 3:** Pros and cons of exosome isolation techniques.

Isolation techniques	Sample size	Equipment required	Separation time	Morphology	Concentration	Purity	References
Ultracentrifugation	Large (≥10 mL)	Ultracentrifuge	Long (4–24 h)	Intact but may deform	Moderate	Moderate	[38–40]
Ultrafiltration	Small–moderate (1–10 mL)	Filtration units (MWCO devices)	Moderate (1–4 h)	May deform vesicles	Moderate	Low‐moderate	[39, 41]
Precipitation	Moderate (≥250 µL)	Kits (e.g., PEG–based reagents)	Short (30 min‐ 2 h)	Aggregates are common	High	Low	[39, 42, 43]
Size‐exclusion chromatography	Small–moderate (0.5–5 mL)	HPLC/FPLC system	Moderate (1–2 h)	Intact vesicles	Low‐moderate	High	[39, 44]
Immunoaffinity capture	Small (as low as 100 µL)	Antibody‐coated beads/microplates	Short‐Moderate (30‐ 90 min)	Highly specific	Low	Very high	[39, 45, 46]
Microfluidic system	Small (10–100 µL; up to 10 mL)	Microfluidic chip	Short (min)	Preserved	High	High	[47–50]

#### 3.1.1. Ultracentrifugation

Ultracentrifugation, which was long considered the gold standard for exosomal isolation, owing to its precedence and widespread adoption, utilizes high centrifugal forces to separate EVs based on differences in size and density [39]. Exosomes in nearly 73.9% of studies are isolated by ultracentrifugation, followed by size‐exclusion chromatography (SEC) (19.6%) and kit‐based methods (4.3%) [52]. This method can be divided into two types: differential ultracentrifugation (DUC) and density gradient ultracentrifugation.

DUC is the most used technique for exosomal isolation (Figure 3). Its principle includes DUC involving sequential centrifugation steps at increasing centrifugal forces. Initially, cells and cell debris are separated at low‐speed centrifugation around 300:2000 ×*g*, respectively. Protein aggregates and apoptotic bodies are eliminated at a slightly higher speed of around 10,000 ×*g*; in contrast, to isolate exosomes, the speed increases dramatically to reach between 100,000 and 120,000 ×*g* [39].

**Figure 3:**
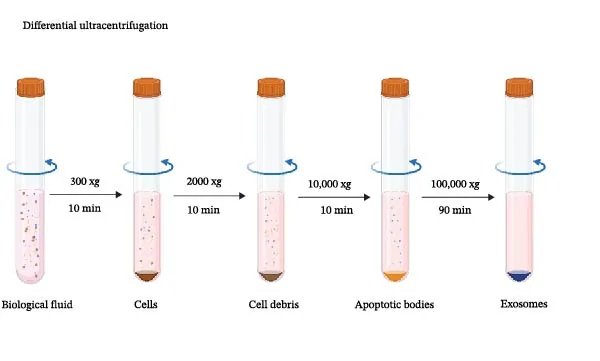
Ultracentrifugation method for isolating exosomes from biofluids. This figure was created by the authors with BioRender.com.

This technique is popular due to its simplicity and low operational costs, but it produces samples of low purity and may result in exosomal membrane damage [51, 53].

Density gradient ultracentrifugation is an enhanced isolation method that integrates DUC with density gradient ultracentrifugation [39, 54]. Like DUC, this technique allows particles to form distinct zones as they deposit at specific positions in a gradient under a specific centrifugal force (Figure 4). However, it employs a preconstructed density gradient in a centrifuge tube, typically made of sucrose or iodixanol [39].

**Figure 4:**
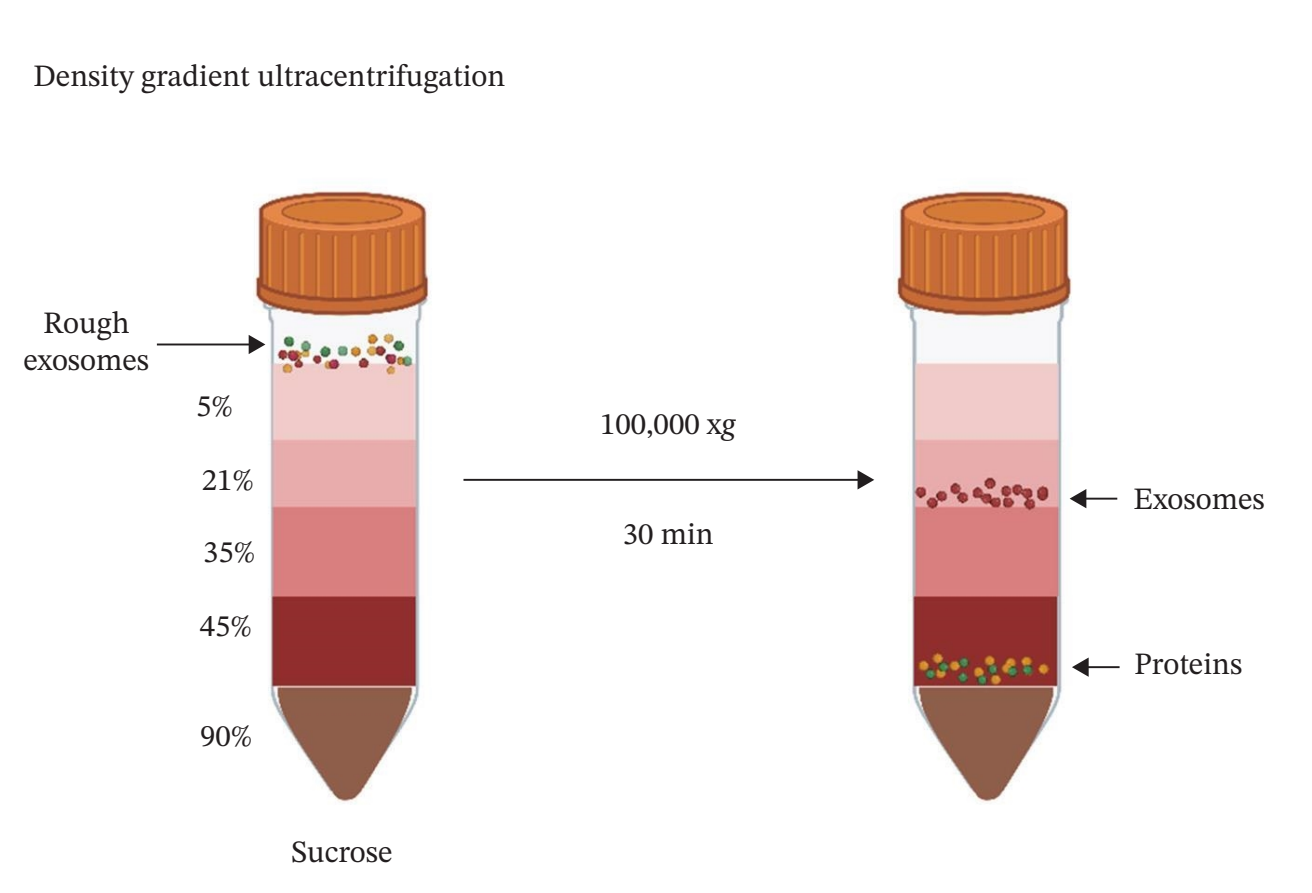
Density gradient ultracentrifugation using different concentrations of sucrose. This figure was created by the authors with BioRender.com.

Exosomes in sucrose solutions have a density that ranges between 1.13 and 1.19 g/mL [54]. Density gradient ultracentrifugation offers purer isolation, preserves the morphological integrity of exosomes, and maintains their biological activity compared to those isolated through DUC [40].

#### 3.1.2. Ultrafiltration

Ultrafiltration is a technique that isolates exosomes based on their size and molecular weight differences using pressure to push sample fluid through porous membranes. Depending on the size of the pores, smaller particles pass through while larger molecules remain behind. It employs ultrafine nanofiltration membranes with varying molecular weight cut‐off values (MWCO) of 10, 50, and 100 kDa [38]. The procedure is faster than ultracentrifugation; nevertheless, the pressure utilized may induce exosome damage due to shear stress, exosome loss owing to membrane adhesion, and membrane clogging due to particle aggregation, which may reduce the exosome yield and increase processing time [41].

There are several types of ultrafiltration: centrifugal ultrafiltration, sequential filtration, tandem filtration, and tangential flow filtration (TFF). In centrifugal ultrafiltration (Figure 5), it uses centrifugal force to drive the sample through a membrane with a defined MWCO, centrifugation forces small molecules through the membrane, and the exosomes are retained above the membrane.

**Figure 5:**
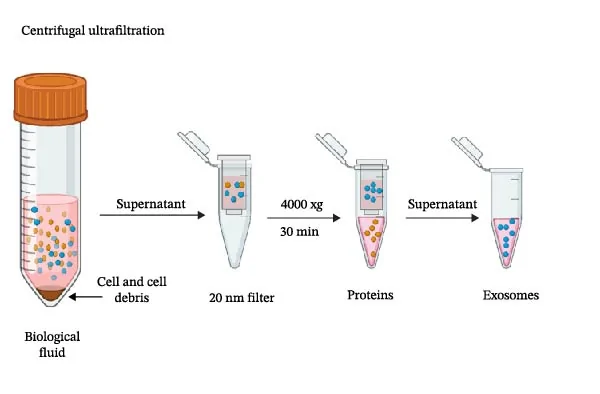
Centrifugal ultrafiltration is a membrane‐based separation technique that uses centrifugal force to concentrate, purify, or fractionate biological samples based on molecular size. The sample is placed in a centrifugal filter device containing a semi‐permeable membrane with a defined molecular weight cut‐off (MWCO). Small proteins and salts pass through the membrane, while larger molecules including exosomes are retained above the membrane. This figure was created by the authors with BioRender.com.

Tandem filtration (Figure 6) combines numerous filters in a single syringe with pore sizes of 30 and 200 nm, while sequential filtration involves multiple rounds of filtering, typically three steps each with a different molecular weight cutoff (Figure 7). Cells and cell debris are filtered in the first phase using membrane filters with 0.1, 0.22, and 0.45 μm pore diameters, free proteins are eliminated, and exosomes are separated using filters with suitable pore diameters, which have molecular weight cutoffs ranging from 5 to 100 kDa. The sample content is forced through a nanoporous membrane that is attached inside a tube by centrifugal force as the membrane is rotated within a tube [55, 56].

**Figure 6:**
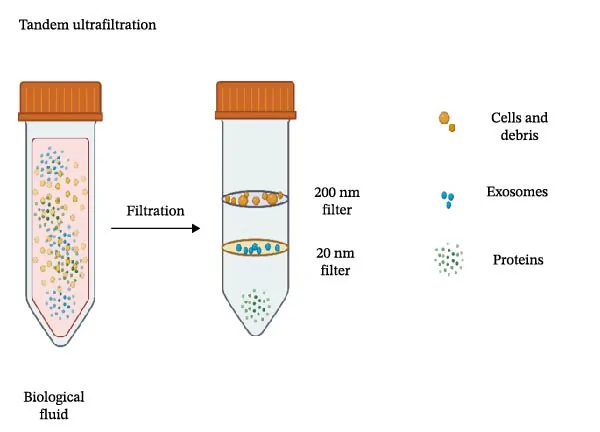
Tandem filtration method for exosome isolation combines numerous filters in a single syringe. This figure was created by the authors with BioRender.com.

**Figure 7:**
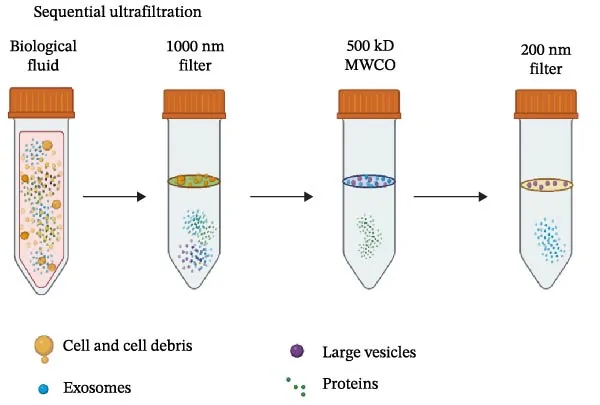
Sequential filtration involves multiple rounds of filtering, each with a different molecular weight cutoff. This figure was created by the authors with BioRender.com.

To optimize the process, a cross‐flow or TFF system was developed using two membranes with pore sizes of 30 and 200 nm (Figure 8), connected to a peristaltic pump that induces continuous circulation of the sample tangentially to the membrane rather than applying pressure orthogonally through the membrane, minimizing membrane clogging, and allows efficient one‐step isolation of exosomes compared to conventional direct filtration [57, 58].

**Figure 8:**
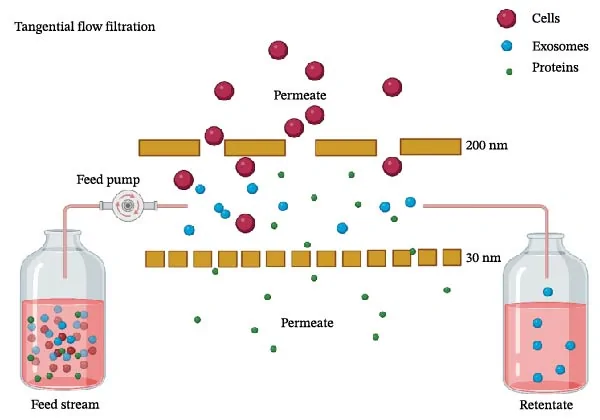
Tangential flow filtration, the feed stream flows parallel to the membrane under hydrodynamic pump force. There are two types of membranes used: microfiltration membrane with pore size of 200 nm permitting cells and cell debris to cross through and ultrafiltration membrane of 30 nm to isolate proteins. Based on the size of the molecules, retentate is the molecules that do not cross the membrane, while the permeate is the molecules that pass through the membrane. This figure was created by the authors with BioRender.com.

The limitations of this method include exosome damage and contamination by solution components that are smaller in size than the pores of the filters. Moreover, some exosomes may be absorbed into the membrane, meaning that some exosomes would be lost, which is important for extracting tiny amounts of biological fluids. Compared to ultracentrifugation methods, TFF is gentler to the sample and can process larger quantities of fluid with improved reproducibility.

#### 3.1.3. Precipitation

Polymer precipitation is a commonly used technique for exosome isolation based on their solubility (Figure 9). Polymer precipitation can be classified into charge methods and hydrophilic polymer precipitation. Precipitation methods isolate exosomes by neutralizing their surface charge or removing their hydration layer, forcing them to clump together and sediment easily during low‐speed centrifugation [39].

**Figure 9:**
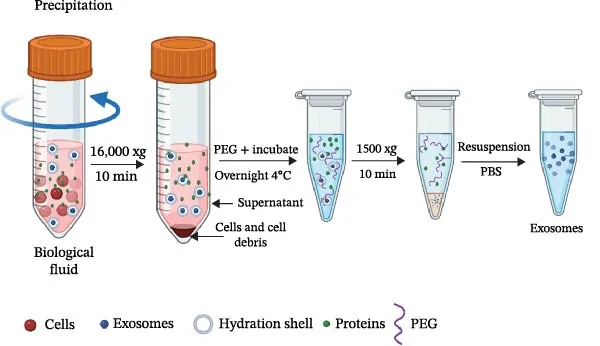
Precipitation method by PEG to isolate exosomes from biological fluid samples. This figure was created by the authors with BioRender.com.

Precipitation techniques rely on several main precipitating agents, such as protamine, sodium acetate, and polyethene glycol (PEG). Exosomes and EVs carry a negative charge due to phosphatidylserine on their surface, allowing positively charged protamine to bind and aggregate with the exosomes. One of the primary issues with this method is that protamine binds strongly to negatively charged exosomes, and after isolation, it remains attached, reducing the purity of the exosomes. Sodium acetate, the organic salt, neutralizes the negative charge of the exosomes and induces aggregation by salting out through the hydrophobic effect [39, 42]. Precipitation using sodium acetate has been reported to yield approximately twofold more total protein than conventional ultracentrifugation under the conditions tested [42]; however, this reflects total protein recovery rather than exosome purity, since precipitation methods, including salting‐out, are prone to co‐precipitating nonexosomal proteins.

Among various hydrophilic polymers used is the highly hydrophilic PEG due to its nontoxic nature, high solubility in water and organic solvents, and strong thermal stability [39]. At a specific concentration, PEG interacts with the water molecules surrounding the exosomes, creating a hydrophobic microenvironment, resulting in exosome precipitation [39]. All precipitation techniques utilize centrifugation to allow the aggregates to settle, but they use simpler devices and require significantly less time compared to ultracentrifugation. Additionally, compared to ultrafiltration, they can accommodate larger volumes of sample [39, 43].

### 3.2. Advanced Exosome Isolation Techniques

While conventional methods, such as ultrafiltration and ultracentrifugation, remain widely used, there is a growing demand for more advanced and efficient methods of exosome isolation. These advanced methods provide higher specificity, yield, and purity, thereby overcoming the limitations of traditional techniques, such as SEC, immunoaffinity capture, and microfluidics‐based techniques (MF) [39].

#### 3.2.1. SEC

The SEC is an isolation technique that relies on the size of the exosomes rather than their molecular weight. SEC employs a solid‐phase medium consisting of porous gel, usually made of sephadex, sepharose, sephacryl, or biogel, filled with agarose beads [39]. When passing a solution through a column consisting of porous beads, molecules can be separated according to size. While particles with radii smaller than that of the pores of the beads enter the pores for longer traffic distance, larger particles, which cannot enter the pores move directly around the beads. This causes particles with different sizes to exhibit different retention times and therefore facilitate size‐based separation (Figure 10). However, quick centrifugation at the beginning of the process is necessary to remove debris, and ultrafiltration is performed to concentrate the sample. SEC cannot be used for large volumes; nonetheless, it is an easy‐to‐operate technique that achieves efficient recovery of exosomes while maintaining biological activity [39].

**Figure 10:**
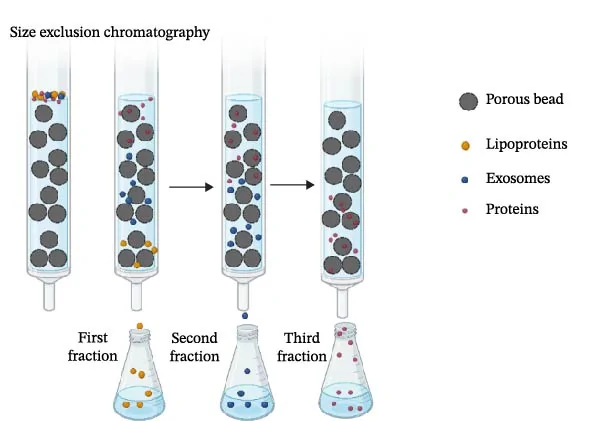
Size exclusion chromatograph (SEC) to isolate exosomes from biological fluid samples. This figure was created by the authors with BioRender.com.

Combining traditional methods like UC with SEC enhances the process compared to using each method independently and increases the overall yield [59, 60]. SEC is more effective at removing small contaminants from body fluids, such as lipoproteins and albumins, which are often present in UC results [44].

#### 3.2.2. Immunoaffinity Capture

Immunoaffinity‐based techniques were designed to overcome the specificity limitations of other methods and produce exosomes with high purity using antibodies and specific affinity ligands [38]. Exosomes are rich in proteins and receptors on their surface, facilitating immunoaffinity‐based isolation through targeted interactions between surface proteins and the immobilized corresponding antibodies or ligands (Figure 11). Exosomes are plentiful in surface proteins, such as transport proteins (GTPases, annexins, and flotillin), vesicle‐forming proteins (Alix and TSG 101), heat shock proteins (Hsc70 and Hsp90), four types of transmembrane proteins called tetraspanins (CD9, CD63, CD81, and CD82), and phospholipase and lipid‐associated proteins, which can serve as biomarkers for binding to the ligands [39, 45].

**Figure 11:**
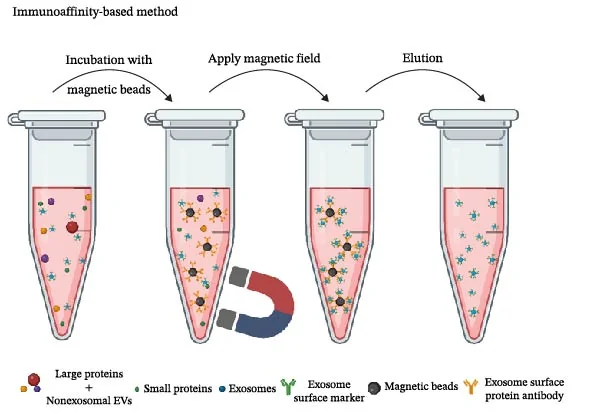
Immunoaffinity‐based method. Magnetic beads coated with antibodies against receptor proteins on the membrane surface of exosomes. This figure was created by the authors with BioRender.com.

Ligands complementary to the membrane surface biomarkers are immobilized into solid materials, such as magnetic beads or polymeric materials. Immunomagnetic beads are the most dominant in this technique, as they utilize a simple external magnetic field. In the presence of the external magnetic field, the magnetic beads bound to the exosomes precipitate and separate when the field is deactivated. This technique provides great flexibility for exosome enrichment, as different proteins can be used for binding [39]. Immunomagnetic beads are considered the new standard protocol for exosome isolation because they are highly specific, easy to operate, and offer high purity [46].

#### 3.2.3. MF

Owing to the physiochemical and biochemical features of exosomes at the microscale, microfabrication technologies have offered a valuable opportunity to develop lab‐on‐chip‐type microfluidic systems for efficient exosome isolation [47, 61, 62].

Microfluidics manipulates small amounts of fluids within microscale channels [63]. This tool reduces sample and reagent consumption, provides higher resolution and sensitivity, and significantly shortens analysis time while improving efficiency. There are currently two types of microfluidic‐based exosome‐separation technologies: size‐based microfluidics and immunoaffinity‐based microfluidics.

##### 3.2.3.1. Size‐Based Microfluidic Separation Techniques

The first size‐based microfluidic system is the exosome total isolation chip (ExoTIC). In this system, samples up to 10 mL are passed through a 0.22 µm nanoporous filter using a syringe pump (Figure 12). The filter retains small EVs (including exosomes), while larger particles pass through. Afterwards, PBS is flushed through the same inlet to wash and recover the exosomes in a small volume of about 200 µL [48]. ExoTIC can isolate exosomes from cell culture media and body fluids such as plasma and urine with minimal damage to their structure. Notably, it can work with very small sample volumes (10–100 µL) and provides 4–1000 times higher than ultracentrifugation in the case of very small input volumes, making it suitable for point‐of‐care clinical applications.

**Figure 12:**
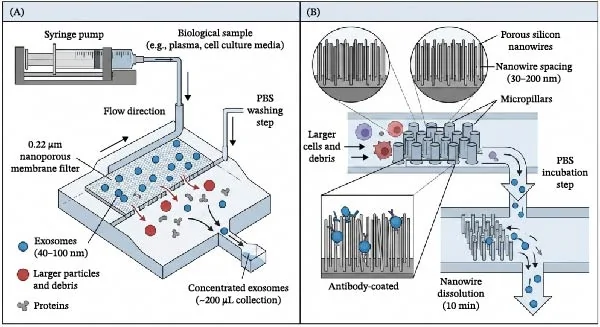
Size‐based microfluidic separation techniques. (A) ExoTIC system (Exosome total isolation chip). (B) Nanowire‐based microfluidic exosome trap. This figure was created by the authors using an AI‐based tool (NanoBanana).

The second system is a nanowire‐based microfluidic exosome trap [49]. This device uses porous silicon nanowires arranged on micropillars. The spacing between nanowires (30–200 nm) is designed to physically capture small EVs, while the larger micropillars remove bigger particles such as cells and cell debris. The isolation efficiency can be further improved by coating the nanowires with exosome‐specific antibodies. This system efficiently isolates exosomes sized 40–100 nm with a recovery rate of about 60%, while allowing proteins and large particles to pass through. The captured exosomes can be gently released by incubating the device in PBS for 10 min, which dissolves the nanowire surface and preserves exosome integrity.

##### 3.2.3.2. Immunoaffinity‐Based Microfluidic Separation Technique

The idea behind the immunomicrofluidic‐based exosome separation devices includes recognition of specific exosome markers by corresponding antibodies immobilized on the surface of the microfluidic chip or magnetic beads (Figure 13). The most common biomarkers that exist on exosomes are tetraspanins, which mainly include CD9, CD63, CD81, and CD147. In 2010, Chen et al. [50] pioneered a microfluidic immunoaffinity apparatus for quick exosome isolation using an anti‐CD63 antibody. The resulting device efficiently isolated exosomes from very small volumes of cell culture medium and serum. Furthermore, by passing 300 µL of lysis buffer through the exosome‐captured microchannel followed by air flushing, the group could easily obtain total RNAs from the captured exosomes.

**Figure 13:**
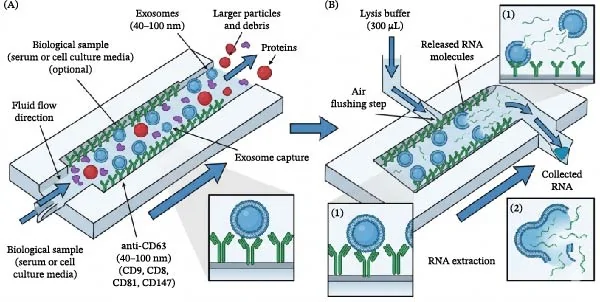
Immunoaffinity‐based microfluidic separation technique. (A) Immunoaffinity capture of exosomes. (B) Exosome lysis and RNA extraction. This figure was created by the authors using an AI‐based tool (NanoBanana). (1) A magnified view of a single exosome (40–100 nm) still intact and specifically bound to the chip surface via the anti‐CD63 antibody, shown immediately before lysis. (2) A magnified view showing the exosome after lysis buffer treatment, the vesicle’s lipid membrane has been disrupted, releasing its internal cargo.

## 4. Exosome Characterization

Exosomes isolated using any of the methods described above should be quantified and characterized in terms of size, morphology, surface charge, density, biomarker expression, yield, and purity. Electron microscopy (SEM/TEM), nanoparticle tracking analysis (NTA), dynamic light scattering (DLS), and flow cytometry (FCM) are examples of commonly used exosome characterization techniques (Figure 14). Exosome characterization methods rely on exosomal morphology and biomarker protein composition. Exosomal biomarker proteins include multitransmembrane proteins (CD9, CD40, CD63, CD81, CD82, and heterotrimeric G proteins GNA), single‐transmembrane proteins (integrins, MHCI and MHCII, heparan sulphate proteoglycans, and transferrin receptor), and lipid‐anchored proteins (CD73 and CD59) [64].

**Figure 14:**
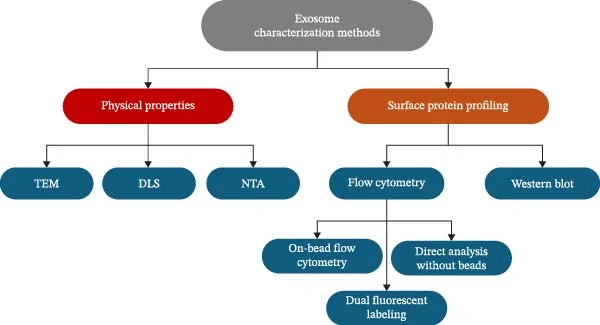
Diagram represents combination of methods to evaluate surface markers, and physical characteristics are necessary for exosome characterization.

### 4.1. Physical Characterization

#### 4.1.1. TEM

The most reliable method for visualizing and analyzing the morphology of exosomes and other EVs is transmission electron microscopy (TEM). Direct observation of these nanoparticles at the nanoscale is made possible by their great resolution, which offers vital information on their size, shape, purity, and integrity [65]. The typical size range of exosomes is 30–150 nm with cup‐shaped structure. As exosomes often show a cup‐shaped appearance that has been so far considered a key morphological feature of EVs. More recently, it has been widely demonstrated that shrinking induced by drying is responsible for this morphology. Cryo‐TEM has in fact shown that EVs are round‐shaped in the vitrified state even if they often appear flattened when they are larger than the ice thickness [66, 67]. Moreover, morphological assessment alone cannot distinguish exosomes from other EV subpopulations or nonvesicular contaminants of similar size. TEM findings should therefore be interpreted alongside complementary characterization approaches, including NTA or DLS for size distribution and concentration, and immunoblotting or FCM for established EV markers (CD9, CD63, CD81, TSG101, and Alix) per MISEV guidelines, to confirm exosome identity and purity.

#### 4.1.2. DLS and Zeta Potential

DLS is a rapid, nondestructive method used to estimate particle size and size distribution based on fluctuations in scattered light caused by Brownian motion using a Zetasizer. Prior to analysis, samples were diluted in sterile PBS to minimize multiple scattering and obtain a suitable particle count. Data were reported as the *Z*‐average diameter and polydispersity index (PDI). Zeta potential was measured using electrophoretic light scattering on the same instrument to provide information on particle surface charge, which is an important parameter for predicting dispersion stability and aggregation behavior.

#### 4.1.3. NTA

Exosomes and other nanoparticles contained in liquid can be physically characterized using the popular analytical technique known as NTA. The NTA is a crucial tool in exosome research because it combines laser light scattering microscopy with video analysis to give a quantitative evaluation of particle size distribution and concentration [68].

Based on the ideas of Brownian motion, NTA is a commonly used method for the quantitative characterization of EVs, especially exosomes. This technique uses a laser beam to illuminate liquid‐suspended particles, and a microscope connected to a video camera records the scattered light from each particle. A comprehensive size distribution profile is then produced by specialized software that tracks each particle’s Brownian motion, giving the particles in the known field of vision and sample volume to determine the particle concentration (usually particles/mL). Fluorescence‐enabled NTA (fl‐NTA), which uses fluorescent labeling to precisely characterize exosome subpopulations based on surface markers, is a noteworthy development [68].

### 4.2. Surface Protein Characterization

#### 4.2.1. FCM

FCM is a commonly used technique to phenotype exosomes by detecting characteristic surface proteins, facilitating use of exosomes in drug delivery, disease research, and diagnostics. It is a potent instrument that enables the simultaneous multiparametric examination of up to thousands of particles each second (physicochemical characteristics including size, shape, and surface biomarkers) [69, 70].

However, conventional instruments have limited sensitivity for nanoscale vesicles (30–200 nm). To address this, exosomes are frequently analyzed using on‐bead FCM, where vesicles are captured onto micrometer‐sized beads and then stained with fluorochrome‐conjugated antibodies against canonical markers such as CD9, CD63, and CD81, as well as disease‐ or cell‐specific antigens. Quantification is typically reported as mean fluorescence intensity (MFI) and/or the percentage of marker‐positive events, enabling semi‐quantitative comparison between samples. More recently, high‐sensitivity (bead‐free) FCM has enabled direct detection of fluorescently labeled exosomes using optimized optics and fluorescence‐based triggering, supporting multiparametric analysis with reduced reliance on bead capture. In addition, dual fluorescent labeling is an effective method for FCM–based exosome characterization and analysis. This method labels various exosome components simultaneously with two separate fluorescent dyes, allowing for more precise identification, measurement, and separation from impurities like micelles or protein aggregates [71, 72].

By taking advantage of their biochemical makeup, dual fluorescence labeling separates exosomes from isolated contaminants: lipophilic dyes (like PKH67 and FM dyes) incorporate into the lipid bilayer, while protein‐specific amine‐reactive dyes (like CFSE) covalently attach to exosomal proteins. True exosomes (dual‐positive), free proteins (protein‐dye only), and lipid aggregates/micelles (lipid‐dye only) can all be identified by combining these colors [72].

Exosomes are subsequently treated with dyes specific to proteins and lipids and then examined using customized FCM. By choosing vesicles that are positive for both dyes, dual‐parameter gating of fluorescence signals verifies the identity of exosomes and enables precise phenotyping of various vesicular subpopulations [73].

#### 4.2.2. WB

Western blotting (WB) is one of the most essential methods for characterizing exosomes. It is frequently used to find protein markers that attest to the existence and purity of isolated exosomes. It offers qualitative and semi‐quantitative data on exosomal proteins, which helps differentiate exosomes from other EVs or contaminants [74].

SDS‐PAGE and immunoblotting are used to determine isolated exosomes in this approach. The WB analysis of exosomes is based on common biomarker receptor proteins expressed and localized on the exosomal membrane, such as CD9, CD40, CD63, CD81, and CD151, and internalized proteins such as tumor susceptibility gene 101 protein (Tsg101), Hsp70, Hsp90, and Alix [75]. In this method, proteins are separated through gel electrophoresis, transported to a nitrocellulose membrane, and probed using certain antibodies. Then, immunoreactive bands will be visualized with an imaging system by comparison with known antigen‐positive and antigen‐negative control samples and a molecular weight ladder (Figure 15). However, no specific protein biomarker for exosomes has been established to ensure the isolated sample contains only exosomes. Altogether, a combination of techniques is needed to detect and characterize exosomes, including their purity, quantification, size, and morphology.

**Figure 15:**
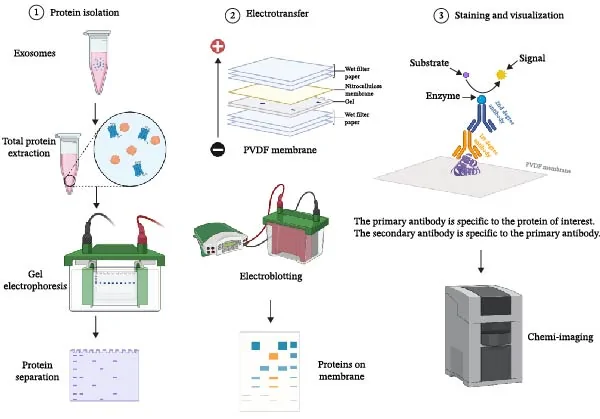
Characterization of exosomal proteins using Western blotting technique. This figure was created by the authors with Biorender.com.

## 5. Engineering and Modification of Exosomes

Exosomes secreted by cells hold significant potential in therapeutic applications due to their low immunogenicity, capacity to target different cells and tissues via the bloodstream, biocompatibility, and ability to cross biological barriers such as the BBB [76]. However, some limitations exist, such as a low quantity of exosomes secreted by the cells, heterogeneity in their content and biological factors, and a short circulation half‐life [76]. These constraints have led scientists and researchers to modify exosomes or engineer their surfaces or load them with different agents such as drugs, siRNA, and CRISPR/Cas9 as shown in Figure 16, using various techniques to improve drug delivery efficiency, enhance targeting of cells and tissues, and extend their circulating half‐life.

**Figure 16:**
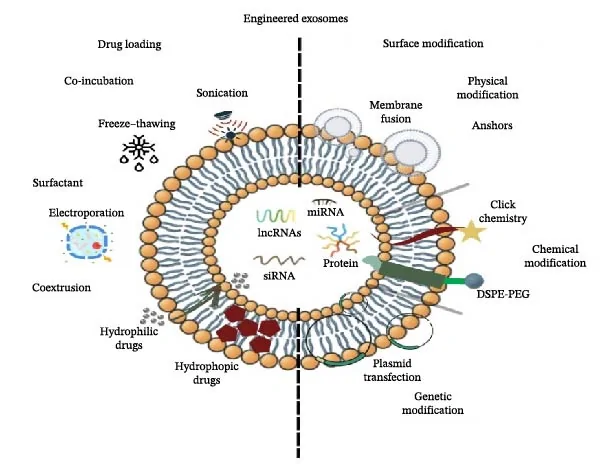
Strategies for engineering exosomes, including surface modifications via physical, chemical, or genetic approaches and drug loading methods like electroporation, sonication. This figure was created by the authors using an AI‐based tool (NanoBanana).

### 5.1. Surface Modification for Targeting

Surface modification plays a vital role in exosome engineering due to its simplicity, convenience, and benefits, such as increased targeting efficiency and prolonged circulation [76]. There are different approaches to surface modification, including physical, chemical, and genetic methods (Table 4).

**Table 4:** Represents the advantages and disadvantages of each surface modification.

Surface modification		Advantages	Disadvantages	References
Physical modification	Membrane fusion	Enhancing exosome membrane properties to boost loading efficiency	Exosome activity weakening	[77, 78]
	Anchors	Enhanced targeting, traceability	Complicated operation, lipids tend to aggregate in aqueous environments	[79, 80]
Chemical modification	Click chemistry	Simple, efficient, and optimized targeting	Potential membrane protein remodeling	[81, 82]
	PEGylation	Prolonging the cycle of exosomes in vivo	Toxic reagents may be required	[83, 84]
Genetic modification	Transfection	Preserved exosome integrity and function	Complex, costly, and insufficiently efficient transfection	[85, 86]

#### 5.1.1. Physical Modification

Physical modification involves altering the exosomal surface using physical forces based on pH differences, freeze–thaw cycles, extrusion, PEG, and natural incubation to promote spontaneous fusion of exosome membranes [76]. The membrane bending modulus increases in acidic environments due to pH‐induced effects, prompting a rearrangement of lipid polar groups. This structural change may influence the energy dynamics of membrane fusion [87, 88].

During freeze–thaw cycles, water expansion within the lipid bilayer changes membrane properties such as fluidity, curvature, and lipid head charge. These alterations enhance membrane interactions and facilitate fusion upon thawing, as observed in experiments aimed at reducing immunogenicity and improving colloidal stability. The findings suggest a novel way to transform exosomes into hybrid nanocarriers, enabling efficient delivery of hydrophobic lipids and hydrophilic cargo to target cells via membrane fusion [89].

Through natural incubation, membranes can undergo spontaneous fusion through their inherent electrostatic and hydrophobic interactions [90]. Membrane fusion increases the size of the exosomes’ membranes, allowing for higher drug loading and macromolecular cargo, reducing immunogenicity, enhancing colloidal stability, and increasing exosome half‐life in blood and target cell uptake [76].

#### 5.1.2. Chemical Modification

Chemical methods enable functional groups to bind to the exosomal membrane through covalent or noncovalent interactions without disrupting membrane integrity [76]. Covalent bonds such as those formed by click chemistry and PEGylation create stable attachments between molecules and the membranes.

Click chemistry can be used to make modifications and changes in exosomes’ surface by attaching targeting ligands, therapeutic agents, and imaging components [91, 92]. This strategy highly depends on several selective reactions between complementary functional groups, allowing modification under mild, biologically compatible conditions while minimizing interference with the native exosome structure and function [91]. Click chemistry can increase and improve the targeting capability of exosomes, their stability, cellular uptake, and delivery to the targeted cells, making the engineered exosomes more suitable as a drug‐delivery system [91, 93].

PEGylation involves attaching polyethylene glycol (PEG) to the exosome surface to overcome their short circulation half‐life [76]. While covalent modifications provide stability, they may require toxic catalysts or conditions, necessitating careful safety assessments for biomedical use. Noncovalent modification methods, such as receptor–ligand binding, electrostatic interactions, and hydrophobic insertion, can also optimize exosome functions [94]. Electrostatic interactions enable binding between cationic targeting moieties and negatively charged groups on membranes, while lipid bilayer structures allow direct membrane insertion through hydrophobic interactions [95, 96].

#### 5.1.3. Genetic Modification

Genetic modification involves transfecting parental cells with plasmid DNA or mRNA encoding genes for specific targeted components (such as antibodies, peptides, and receptors) fused to exosomal membrane proteins like tetraspanins and lysosome‐associated membrane proteins to enable site‐specific insertions, deletions, or modifications in the genome, thereby enhancing exosomal activity. The genetically modified cells produce exosomes containing these desired components through normal biogenesis, maintaining their integrity and function [87].

### 5.2. Drug Loading

Exosomes naturally carry a broad range of bioactive molecules, making them promising carriers for targeted therapy. They have been successfully used to deliver various therapeutic agents, including small‐molecule drugs, RNA–based therapies, and gene‐editing tools. Exosomes can be loaded with therapeutic agents either before their isolation (preloading) or after isolation (postloading), using a variety of physical or chemical techniques. Exosome‐loaded nanoparticle systems have demonstrated therapeutic benefits across diverse conditions, including neurodegeneration [97], periodontitis [98], and inflammatory skin disease [99].

In preloading approaches, exogenous cargo is introduced into donor cells prior to exosome isolation, allowing the packaging of the therapeutic material during exosome biogenesis. For instance, CRISPR/Cas9 plasmids have been successfully incorporated into exosomes through the transfection of donor cells using standard chemical reagents [100]. While postloading techniques involve loading therapeutic agents into exosomes after they have been isolated. Several physical strategies have been explored to enhance loading efficiency. Among these techniques, electroporation remains the most widely used for its relatively high loading efficiency and scalability. Applying electroporation to load a variety of therapeutic cargos to exosomes using short electrical pulses in order to increase the exosomal lipid membrane permeability, allowing them to enter, then the membrane recovers after cutting the pulse. Electroporation is utilized to efficiently load small molecule drugs, and nucleic acids such as siRNAs, miRNAs, and others, also, proteins can be loaded [101]. However, there are several factors, such as voltage, the duration of the pulse, exosome concentration, cargo‐to‐exosome ratio, and buffer composition. Additionally, the loading of nucleic acids is complicated by the precipitation and aggregation of the cargo [101, 102]. However, concerns remain regarding exosome membrane integrity and aggregation.

Sonication is another exogenous strategy that is applied to successfully insert different types of cargos such as small‐molecule drugs, nucleic acids, and proteins. An ultrasound probe is used to form mechanical shear forces that disrupt or deform the lipid membrane of exosomes for a temporary moment to allow the molecules to enter inside; after that the membrane of exosomes recover and reorganize, therefore entrapping the cargo inside [103]. It provides relatively high loading efficiency and can support sustained cargo release. Nevertheless, overuse of sonication can change and alter the morphology of exosomes, affect the integrity of the membrane, aggregation will increase and the efficiency of loading will be affected. Hence, sonication is a sensitive approach in cargo loading [104–107].

Another innovative approach involved mechanical extrusion to coat gold nanoparticles with exosome membranes. This biomimetic method retained the orientation and composition of exosomal membrane proteins and lipids, successfully reducing macrophage uptake and enhancing cellular targeting, although overall yield remained a limitation [108].

Beyond direct cargo loading, exosomes can also be engineered for sustained local delivery by embedding them within injectable or in‐situ‐forming hydrogels, which protect the vesicles from rapid clearance and provide controlled release at the target site. For example, an injectable hyaluronic acid hydrogel loaded with MSC‐derived exosomes was shown to enhance the healing of chronic wounds by prolonging local exosome retention and bioactivity [109]. Similarly, a hypoxia‐induced, exosome‐loaded hydrogel formed in situ improved diabetic wound healing by combining sustained exosome release with a hypoxia‐mimicking microenvironment that promoted angiogenesis [110].

## 6. Future Considerations of Stem Cell Exosomes

### 6.1. Advantages of Exosome‐Based Therapies

In the context of regenerative medicine, stem cell‐derived exosomes have emerged as promising therapeutic tools due to their potent paracrine signaling capacity, ability to modulate immune responses, and support for tissue repair and regeneration [111, 112].

One major advantage of exosome‐based therapy over traditional cell‐based therapy is safety. Exosomes are acellular vesicles that lack replicative potential, which substantially reduces the risk of tumorigenesis associated with whole‐cell transplantation, particularly with pluripotent stem cells [113]. They also avoid complications such as vascular occlusion or inappropriate differentiation. Moreover, exosomes can be stored, sterilized, and standardized more easily than live cells, offering improved stability and shelflife, which are crucial for large‐scale clinical applications [114].

Another significant advantage lies in targeted delivery and immunomodulation. Exosomes possess inherent homing capabilities, often mediated by surface adhesion molecules and integrins, enabling them to reach injured tissues or specific cell populations [115]. MSC‐derived exosomes, for instance, have shown robust immunosuppressive and anti‐inflammatory properties in models of autoimmune disease, myocardial infarction, and neuroinflammation [116]. In addition, the lipid bilayer structure of exosomes protects their cargo from enzymatic degradation in the extracellular environment, enhancing bioavailability and therapeutic efficacy [117].

Furthermore, exosome therapy allows for bioengineering and customization. Through techniques such as electroporation or transfection, therapeutic RNA, proteins, or drugs can be loaded into exosomes, creating multifunctional delivery systems that surpass the natural potential of stem cells alone [118]. These attributes position exosome‐based therapies as scalable, customizable, and clinically safer alternatives to stem cell transplantation.

### 6.2. Limitations of Exosome‐Based Therapies

Despite their many advantages, exosome‐based therapies also exhibit inherent limitations when compared to conventional stem cell therapies. One of the primary drawbacks is the limited regenerative capacity. While exosomes can modulate signaling pathways and promote endogenous repair, they cannot fully recapitulate the complex regenerative functions provided by viable stem cells, such as tissue engraftment, differentiation, and extracellular matrix remodeling [119]. In cases where structural tissue replacement is necessary, whole‐cell transplantation remains superior.

Another critical limitation is the variability in exosome content and function. Exosome cargo is highly dependent on the physiological state of the parent stem cells, the culture conditions, and the methods used for isolation and purification. This variability complicates quality control and consistency between batches. Batch‐to‐batch cargo heterogeneity is not merely a quality‐control inconvenience but remains one of the principal unresolved barriers to reproducible clinical translation of exosome‐based therapeutics and is not yet fully addressed by current isolation or characterization standards [120].

Moreover, different isolation techniques such as SEC, ultracentrifugation, and polymer‐based precipitation yield exosomes of varying purity and biological activity, impacting reproducibility across studies [121].

The lack of long‐term engraftment or integration also limits exosome therapy. While this feature improves safety by reducing the risk of unwanted proliferation, it also means that the therapeutic effects are often transient, necessitating repeated administration to achieve sustained benefit [122]. This could pose logistical and economic burdens in chronic diseases. Additionally, dosage and pharmacokinetics remain poorly defined. Unlike traditional small‐molecule drugs or even stem cells, the optimal dose, frequency, and route of exosome administration are still under investigation. Exosomes are rapidly cleared from circulation by the mononuclear phagocyte system, particularly in the liver and spleen, which may limit their systemic therapeutic impact [123].

### 6.3. Immunogenicity and Safety

A key concern in any biological therapy is immunogenicity, and exosome‐based therapeutics, while generally considered safe, are not entirely free of immunological risk. Exosomes derived from allogeneic stem cells can carry surface antigens, including MHC molecules and co‐stimulatory proteins, which may elicit immune recognition and rejection in some recipients. However, their immunogenicity is significantly lower compared to whole‐cell therapies, particularly because exosomes do not replicate or persist long‐term in host tissues [124].

Another safety consideration is the potential for off‐target effects or unwanted cargo delivery. Because exosomes can cross biological barriers, including the BBB, and distribute systemically, they may interact with nontarget tissues, possibly delivering unwanted RNA, proteins, or bioactive molecules. This is particularly relevant in the case of poorly characterized or inadequately purified exosome preparations [125]. To address these concerns, efforts are underway to standardize manufacturing protocols, including the use of GMP‐compliant cell sources, rigorous purification steps, and characterization of exosome surface markers and content. Moreover, advances in exosome engineering may allow the development of surface‐modified exosomes with reduced immunogenicity and enhanced targeting precision [95].

## 7. Conclusion

Exosomes, particularly those derived from stem cells, have demonstrated significant potential as targeted therapeutic agents in regenerative medicine. Their molecular cargo plays critical roles in regulating gene expression, modulating immune responses, and promoting tissue repair. Advances in exosome isolation and characterization techniques have further enhanced our understanding of their composition and biological functions.

However, several challenges remain, including the need for standardized production protocols, scalable manufacturing processes, and comprehensive cargo profiling to ensure consistency and reproducibility. Additionally, further preclinical and clinical studies are required to fully establish their safety, efficacy, and therapeutic applicability.

Overall, exosomes represent a promising and evolving platform that may contribute to the future of regenerative medicine.

## 8. Limitations

Despite the promising potential of exosomes in regenerative medicine, several important limitations should be acknowledged. One of the main challenges is the lack of standardized methods for their isolation and characterization, which leads to variability in results between studies. In addition, exosomes can differ significantly depending on their cell source and preparation conditions, making it difficult to directly compare findings across the literature.

Another limitation is that the exact mechanisms by which exosomes exert their effects are not yet fully understood, particularly regarding how their cargo is selectively packaged and delivered to target cells. Furthermore, although preclinical results are encouraging, clinical translation is still limited, with few large‐scale human trials available. Issues such as large‐scale production, quality control, and regulatory approval also remain unresolved.

## Author Contributions

Conceptualization and supervision: Yasmine Hosny and Hazem A. El‐Kady. Writing: Yasmine Hosny, Hazem A. El‐Kady, Majdeldin E. Abdelgilil, Hagar W. Mandour, Rahaf Altartoor, Ziad Mustafa Abd El‐Wahab, Sarah Alosman, Soha M. Abdelzaher, and Ibrahim M. Fangary. Revision: Yasmine Hosny and Hazem A. El‐Kady. Drawing the figures: Yasmine Hosny.

## Funding

This research did not receive any specific grant from funding agencies in the public, commercial, or non‐for‐profit sectors.

## Disclosure

All authors have read and agreed to the published version of the manuscript. The authors critically reviewed and validated the output and assumed full responsibility for the final content using an AI‐based tool (NanoBanana).

## Ethics Statement

The authors have nothing to report.

## Consent

The authors have nothing to report.

## Conflicts of Interest

The authors declare no conflicts of interest.

## Data Availability

The data that support the findings of this study are available upon request from the corresponding author.
